# CeO_2_ Nanorods Embedded in Ni(OH)_2_ Matrix for the Non-Enzymatic Detection of Glucose

**DOI:** 10.3390/nano7080205

**Published:** 2017-07-31

**Authors:** Yongjian Li, Panpan Guan, Fucheng Yu, Wei Li, Xiaoling Xie

**Affiliations:** 1 Department of Material Science, Taiyuan University of Technology, Taiyuan 030024, China; Leeyj2015@163.com (Y.L.); guanpanpan@139.com (P.G.); 2 School of Material Science, Lanzhou University of Technology, Lanzhou 730050, China; yufc72@163.com

**Keywords:** cerium oxide, nickel hydroxide, biosensor, glucose, nanocomposite

## Abstract

The electrode based on cerium oxide (CeO_2_) nanorods embedded in nickel hydroxide (Ni(OH)_2_) matrix were prepared and used for detecting glucose non-enzymatically. The materials were characterized by X-ray diffraction, transmission electron microscopy (TEM), and so on. The results indicate that the response of CeO_2_/Ni(OH)_2_ nanocomposite are significantly improved due to the synergetic effect between CeO_2_ and Ni(OH)_2_. The optimum CeO_2_/Ni(OH)_2_ nanocomposite electrode exhibits a detection range from 2 μM to 6.62 mM, a sensitivity of 594 μA mM^−1^ cm^−2^, an estimated detection limit of 1.13 μM, and a response time less than 5 s. In addition, this biosensor also shows good selectivity, long term stability, and accurate measurement in juice on sale.

## 1. Introduction

Glucose biosensors have received considerable attention because of their promising applications in clinical diagnosis, the food industry, and environmental monitoring [1,2,3,4]. Generally, glucose sensors can be classified as either enzymatic glucose sensors or non-enzymatic glucose sensors. Although enzymatic glucose sensors are sensitive and selective, they are limited by the instability of the enzyme, which may be affected by various factors such as temperature, pH, and humidity [5,6,7,8]. Non-enzymatic glucose sensors can overcome the disadvantages of enzymatic glucose sensors. Nevertheless, enzyme-free biosensors usually require suitable sensing materials that can simultaneously catalyze the redox reaction of glucose and rapidly transfer the charges.

Metal oxides (e.g., NiO, CuO) and metal hydroxides (e.g., Ni(OH)_2_, Cu(OH)_2_) possess unique electrocatalytic properties and have been extensively explored for non-enzymatic glucose biosensors in the past ten years. However, the poor intrinsic charge transfer abilities of these oxides or hydroxides impede their application in the electrochemical biosensor, which generally requires a high sensitivity, fast response, and good selectivity [9,10]. An effective way to overcome this limitation is combining these metal oxides or hydroxides with high charge-transporting materials, and the corresponding synergetic effect originating from the combination of components can provide a new interface through which charge and energy transport are significantly enhanced [11].

The high charge-transporting components usually are noble metals [12,13], carbon nanotubes [14,15,16], and graphene [17]. However, only a few studies have been carried out to investigate semiconducting oxides with fast electron-transfer abilities as modification materials (e.g., TiO_2_, ZnO) [18,19,20,21,22]. For example, Gao et al. combined semi-metallic TiO_2_ nanotubes arrays and Ni(OH)_2_ nanoparticles to form a hybrid that could detect glucose with a high sensitivity of 240 μA mM^−1^ cm^−2^, a detection limit of 5.0 μM (S/N = 3), and a quick response time of less than 5 s [9]. Zhou et al. used the electrospinning method to fabricate a CuO/ZnO hierarchical nanocomposite that exhibited an ultrahigh sensitivity of 3066.4 μA mM^−1^ cm^−2^, a linear range of up to 1.6 mM, and a low practical detection limit of 0.21 μM [23]. Given that metal oxides have the advantages of cost effectiveness and simple synthesis, pure metal oxide nanocomposites for application in non-enzymatic biosensor should still be a subject undergoing intense study.

Recently, nanostructured CeO_2_ has been of great interest in electrochemical biosensors because of its unique properties, such as high mechanical strength, high isoelectric point, biocompatibility, and fast electron-transfer ability [24]. However, pristine CeO_2_ is seldom used in non-enzymatic glucose biosensors because it fails to catalyze a surface redox reaction [25,26]. CeO_2_ may be a good modification material for common electrocatalytic materials (e.g., NiO, Ni(OH)_2_) because it has good electronic conductivity, particularly with regard to enhancing their electron-transfer abilities. Using this as an inspiration, the nanorod (NR) structure of CeO_2_ was combined with Ni(OH)_2_ to form a nanocomposite which was used for the first time as a non-enzymatic glucose biosensor.

## 2. Results and Discussion

**Figure 1:**
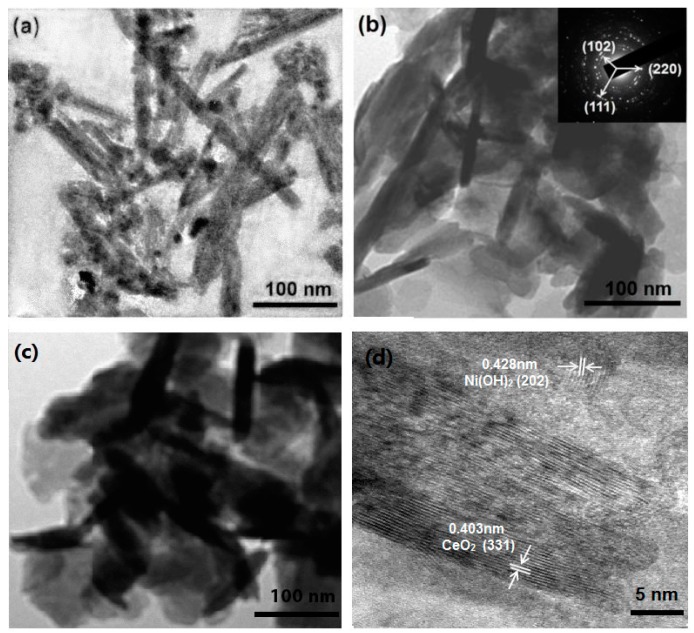
Transmission electron microscopy (TEM) images of (**a**) CeO_2_; (**b**) CeO_2_/Ni(OH)_2_ nanocomposite (Ce:Ni = 0.05:1); and (**c**) CeO_2_/Ni(OH)_2_ nanocomposite (Ce:Ni = 0.5:1); (**d**) High-resolution TEM (HRTEM) image of CeO_2_/Ni(OH)_2_ nanocomposite (Ce:Ni = 0.05:1). Inset is the SAED pattern.

Transmission electron microscopy (TEM) images of CeO_2_ NRs and CeO_2_/Ni(OH)_2_ nanocomposites are shown in Figure 1. The length of CeO_2_ NRs is approximately 100 nm to 200 nm, whereas the diameters of NRs are approximately 10 nm to 20 nm (Figure 1a). Figure 1b clearly shows that CeO_2_ NRs are embedded in the Ni(OH)_2_ matrix. We also investigated the TEM image (Figure 1c) of the nanocomposite with a high Ce:Ni ratio (0.5:1), in which the sample exhibited similar structure to the nanocomposite with a small Ce:Ni ratio (0.05:1). The circles in the selected area electron diffraction (SAED) pattern are attributed to the diffraction planes of Ni(OH)_2_ (102), CeO_2_ (111), and CeO_2_ (220) (inset of Figure 1b), which indicates the polycrystalline structure of the nanocomposites. High-resolution TEM (HRTEM) image of CeO_2_/Ni(OH)_2_ nanocomposite is shown in Figure 1d. The lattice distances are in good agreement with the typical parameters of Ni(OH)_2_ (JCPDS 14-0117) and CeO_2_ (JCPDS 34-0394).

**Figure 2:**
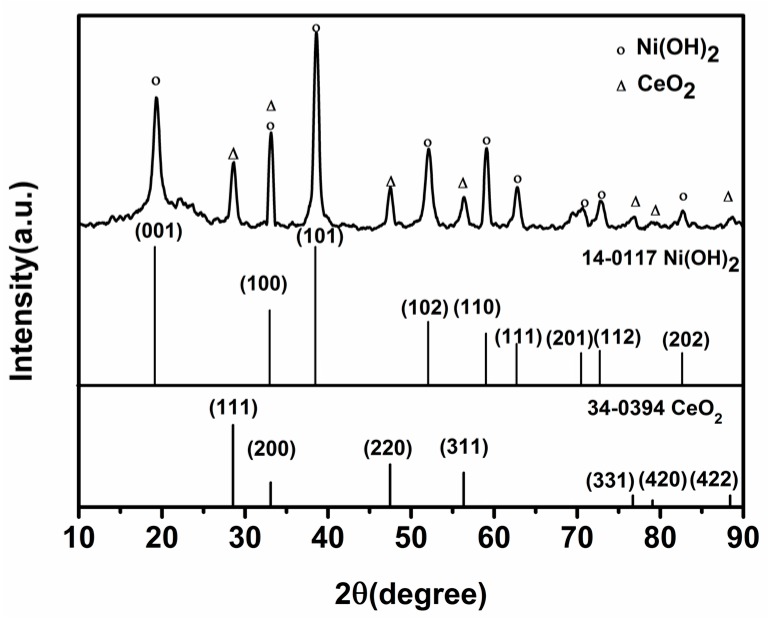
XRD patterns of CeO_2_/Ni(OH)_2_ nanocomposite (Ce:Ni = 0.05:1).

The X-ray diffraction pattern (XRD) of CeO_2_/Ni(OH)_2_ nanocomposite (Ce:Ni = 0.05:1) is shown in Figure 2. The diffraction peaks at 19.26°, 33.06°, 38.54°, 52.10°, 59.05°, 62.73°, 72.74°, 73.13° and 82.61° correspond to the (001), (100), (101), (102), (110), (111), (201), (112) and (202) planes of Ni(OH)_2_, respectively. The appearance of these peaks implies a hexagonal structure of Ni(OH)_2_ (JCPDC 14-0117). The peaks that appeared at 28.55°, 33.08°, 47.48°, 56.33°, 76.70°, 79.07° and 88.41° are related to the (111), (200), (220), (311), (331), (420) and (422) planes of CeO_2_ and indicate a cubic fluorite structure of CeO_2_ (JCPDS 34-0394). 

**Figure 3:**
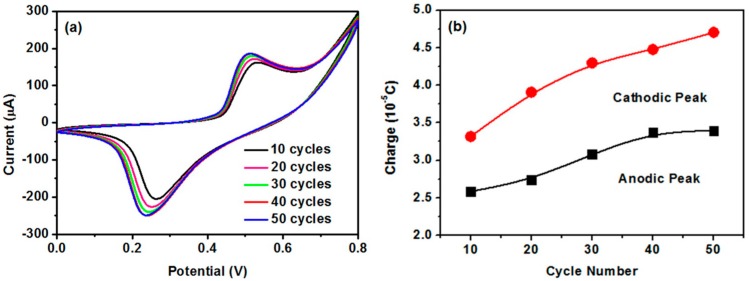
(**a**) CVs of CeO_2_/Ni(OH)_2_/CPE after 10, 20, 30, 40, and 50 consecutive cycles in 0.1 M NaOH solution at 50 mV/s scan rate; (**b**) Plots of charges integrated from anodic and cathodic peaks in CVs versus number of cycles.

The cyclic voltammogram (CV) obtained by continuously cycling the electrode potential between 0 and 0.8 V against Ag/AgCl reference electrode was used to investigate the electrocatalytic activities of the electrodes. CV scanning was repeated until a stationary state was obtained because nickel-based electrodes usually suffer from fouling under repeated cyclic sweeping in CV measurements, which can be attributed to the change in crystal structure [15]. Such a process is shown in Figure 3a, which demonstrates a pair of well-defined anodic and cathodic peaks corresponding to the redox reaction of Ni(II)/Ni(III) [1,12,27,28]. A stationary state was obtained after 40 scan cycles, which indicates that the crystal structures had stabilized. The relation between peak charges integrated from the CVs and scanning cycles is shown in Figure 3b. The charges gradually increased with an increase of cycle number, which indicates that electrocatalytic reactions mainly occurred on or near the surface of Ni(OH)_2_ [1,29].

**Figure 4:**
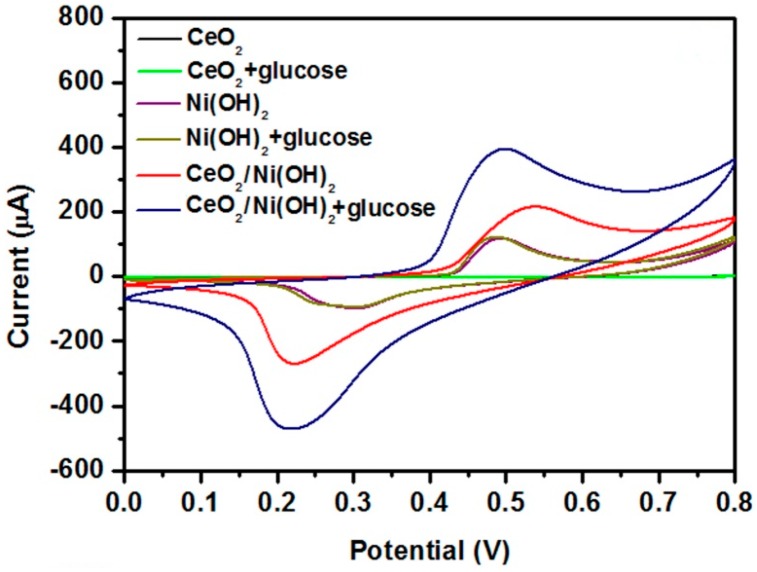
CVs of CeO_2_/CPE, Ni(OH)_2_/CPE, and CeO_2_/Ni(OH)_2_/CPE in the absence and presence of 1 mM glucose in 0.1 M NaOH solution at 50 mV/s scan rate.

**Figure 5:**
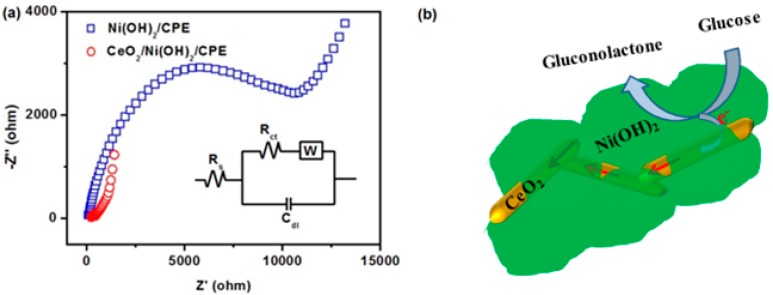
(**a**) Nyquist impedance plots for Ni(OH)_2_/CPE and CeO_2_/Ni(OH)_2_/CPE recorded in 0.1 M KCl solution containing 1 mM Fe[(CN)_6_]^3−/4−^ (1:1); (**b**) Schematic diagram of sensing mechanism of CeO_2_/Ni(OH)_2_ nanocomposite.

Figure 4 shows the electrocatalytic activities of three different electrodes to glucose. Ni(OH)_2_/CPE (carbon paste electrode) exhibited a slight change in peak current after adding 1 mM glucose, in which the magnitude of such change actually increased with an increase of glucose concentration (not shown). By contrast, a clear non-enzymatic response to 1 mM glucose was observed from CeO_2_/Ni(OH)_2_/CPE. The enhanced response of CeO_2_/Ni(OH)_2_/CPE is a reflection of the synergetic effect obtained from the combination of CeO_2_ and Ni(OH)_2_. Pure CeO_2_ component had no electrocatalytic activity; however, it influenced the interfacial electron transfer process which was investigated by electrochemical impedance spectroscopy (Figure 5a). The Randles circuit (inset of Figure 5a), which contains electrolyte resistance (R_s_), charge transfer resistance (R_ct_), double layer capacitance (C_dl_), and Warburg element (W), was used to model these impedance plots. Based on the study of Patil et al. [24], lower R_ct_ values were attributed to an accelerated electron transfer between the modified electrodes and electrolytes. Using ZSimpWin (v3.60) software, the R_ct_ values that best fit the experimental impedance plots were approximately 11,078 and 178 Ω for Ni(OH)_2_/CPE and CeO_2_/Ni(OH)_2_/CPE, respectively, which indicates that CeO_2_/Ni(OH)_2_/CPE has a higher charge transfer ability than Ni(OH)_2_/CPE. Such an enhancement is related to the CeO_2_ NRs, which randomly contact each other in the Ni(OH)_2_ matrix (Figure 5b) and consequently form conductive paths for electron transfer.

The Ni(OH)_2_ component dominated the redox reaction of glucose by means of the Ni(II) /Ni(III) redox couple. The reaction in the blank NaOH solution can be expressed as Equation (1) [9]. After adding glucose, the Ni(III) ion could obtain an electron from the oxidation of glucose and deliver this electron to the electrode, thereby leading to an increase of peak current. The reaction is depicted in Equation (2). 

Ni(OH)_2_ + OH^−^ → NiO(OH) + e^−^ + H_2_O (1)

NiO(OH) + glucose →Ni(OH)_2_ + glucolactone (2)

**Figure 6:**
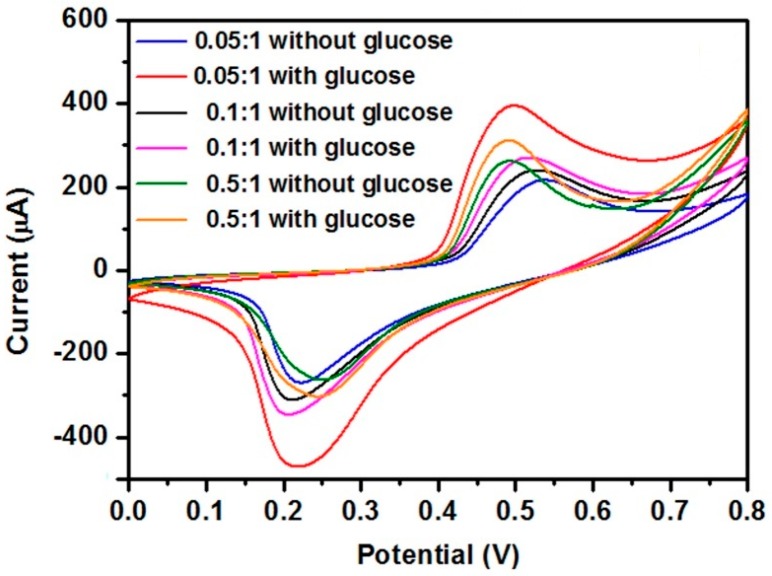
CVs of CeO_2_/Ni(OH)_2_/CPE with different Ce:Ni molar ratios in the absence and presence of 1 mM glucose in 0.1 M NaOH solution at 50 mV/s scan rate.

**Figure 7:**
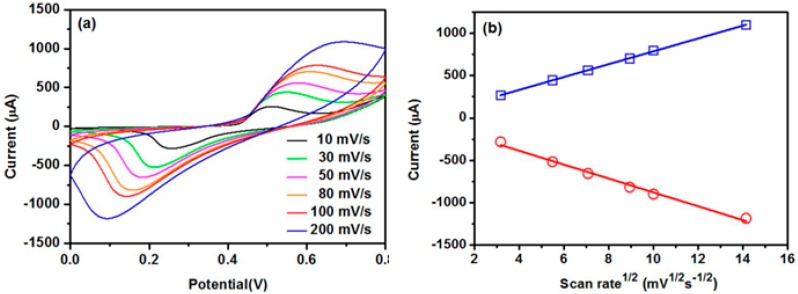
(**a**) CVs of CeO_2_/Ni(OH)_2_/CPE in 0.1 M NaOH solution containing 1 mM glucose at different scan rate; (**b**) Plot of the peak current with square root of scan rate.

Figure 6 is the effect of the molar ratio of cerium to nickel on the sensing response to glucose. The results show that an optimum response can be obtained when the molar ratio is equal to 0.05:1. The influence of the scan rate on the current response is shown in Figure 7a. The magnitude of the peak current increased with the increasing scan rate. The peak current was quite linear to the square root of the scan rate (Figure 7b). Such linearity further confirmed the diffusion-controlled kinetics at the electrode surface. 

**Figure 8:**
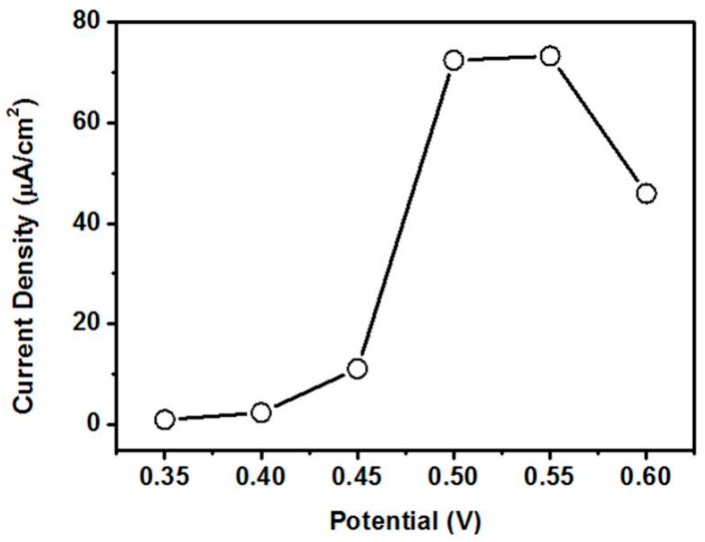
The response current of CeO_2_/Ni(OH)_2_/CPE measured in 0.1 M NaOH for 1 mM glucose at different potentials.

The amperometric response of CeO_2_/Ni(OH)_2_/CPE was further investigated. Finding a suitably applied potential that can obtain maximum catalytic current is necessary because the applied potential intensely influences the amperometric response [4]. Hydrodynamic voltammetry measurement to 1 mM glucose was conducted in 0.1 M NaOH solution to find an optimum applied potential. As shown in Figure 8, CeO_2_/Ni(OH)_2_/CPE demonstrated the highest response current at 0.55 V. The current density drastically declined over this potential. Based on Dung et al. [15], such decrease is possibly attributed to the oxidation of water interfering with the oxidation of glucose or the products of glucose oxidation poisoning the electrode. Therefore, the amperometric response was investigated at 0.55 V based on the results of hydrodynamic voltammetry. 

**Figure 9:**
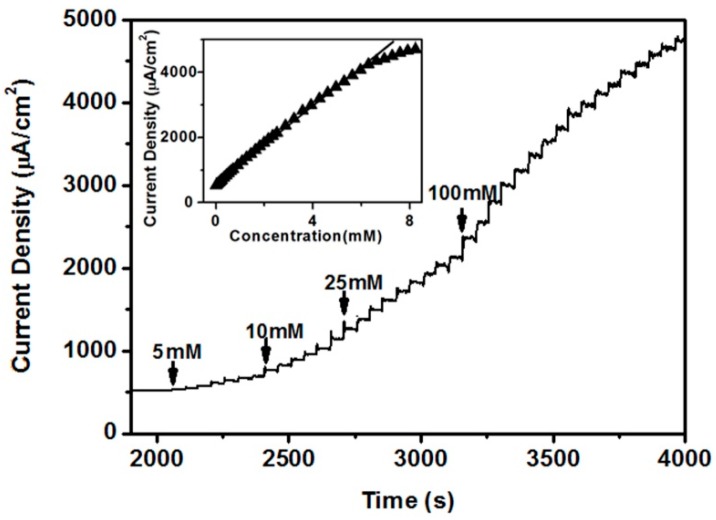
Amperometric response of CeO_2_/Ni(OH)_2_/CPE measured at 0.55 V in 0.1 M NaOH solution with successive addition of glucose. Inset is the plot of current response with glucose concentration.

Figure 9 shows the amperometric response of the CeO_2_/Ni(OH)_2_/CPE with the successive addition of glucose in 0.1 M NaOH solution at 0.55 V. The current increased with the glucose addition because of the good catalytic properties of the CeO_2_/Ni(OH)_2_/CPE. The response time which means that the current reaches 95% of the steady state value was less than 5 s. A calibration curve was constructed from this amperometric response curve (inset of Figure 9). CeO_2_/Ni(OH)_2_/CPE exhibited a good linearity with glucose concentration, ranging from 2 μM to 6.62 mM with a correlation coefficient of 0.998. A high sensitivity of 594 μA mM^−1^ cm^−2^ was obtained from the slope of the calibration curve. Moreover, the limit of detection was 1.13 μM at a signal to noise ratio of 3. A comparison among several typical glucose biosensors reported in the last five years is shown in Table 1. The CeO_2_/Ni(OH)_2_/CPE exhibits superiority, especially with regard to sensitivity, linear range, and detection limit. This superiority is attributed to the synergetic effect by combining CeO_2_ NRs with Ni(OH)_2_, which dramatically increases the charge transfer and electrocatalytic activity of nanocomposite.

**Figure 10:**
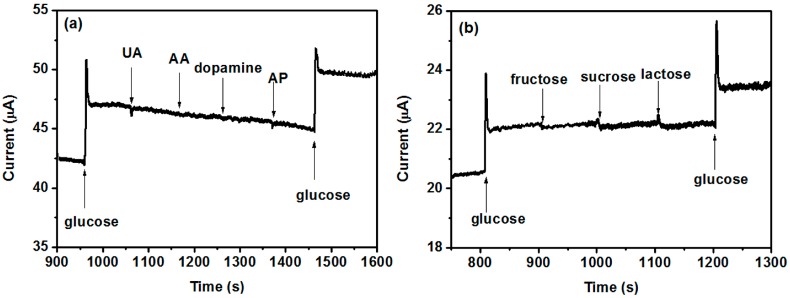
(**a**) Interference test of CeO_2_/Ni(OH)_2_/CPE upon continuous adding 5 mM glucose, 0.1 mM UA, 0.1 mM AA, 0.1 mM dopamine, 0.1 mM AP, and 5 mM glucose at 0.55 V in 0.1 M NaOH solution; (**b**) Interference test of CeO_2_/Ni(OH)_2_/CPE upon continuous adding 5 mM glucose, 0.1 mM fructose, 0.1 mM sucrose, 0.1 mM lactose, and 5 mM glucose at 0.55 V in 0.1 M NaOH solution.

**Figure 11:**
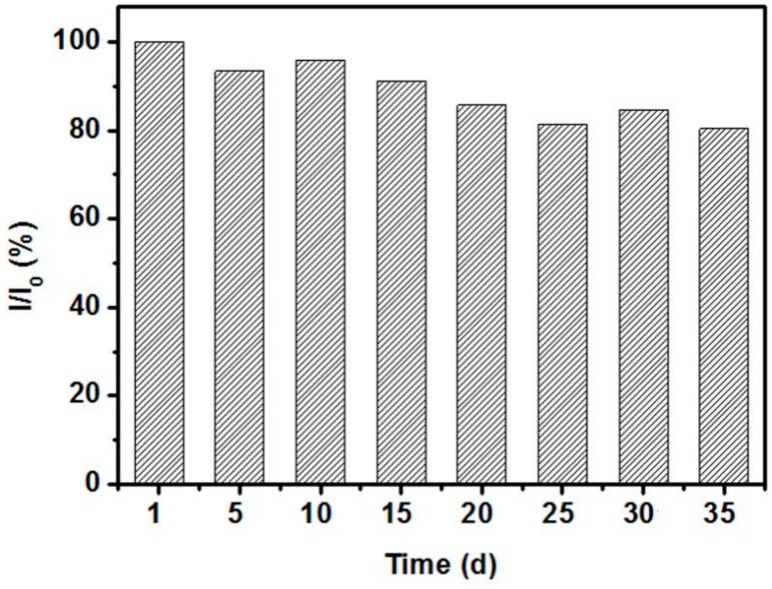
Stability of CeO_2_/Ni(OH)_2_/CPE electrode at ambient conditions for seven weeks using 1 mM glucose in 0.1 M NaOH solution at 0.55 V.

Selectivity is an essential feature of glucose biosensors for practical applications, especially for physiological test. Generally, the concentrations of interfering species such as ascorbic acid (AA), uric acid (UA), 4-acetamidophenol (AP), fructose, and lactose are no more than 0.1 mM in the physiological fluid, whereas the normal level of glucose is approximately 3–8 mM [1,6]. Therefore, the amperometric experiment regarding the selectivity of the sensor was conducted with 5 mM of glucose and 0.1 mM of various interfering species. Figure 10 shows that all these interfering species had negligible responses compared with glucose, which indicates that the CeO_2_/Ni(OH)_2_/CPE fabricated in this work has sufficient selectivity for practical application. The long-term stability of CeO_2_/Ni(OH)_2_/CPE was investigated by determining its current response (I) to 1 mM glucose every five days under ambient conditions for seven weeks, during which the sensor preserved 80.36% of its original value (I_0_), thereby reflecting a good stability of biosensor (Figure 11). 

A kind of juice on sale was chosen as the test-target to investigate the practical application of CeO_2_/Ni(OH)_2_/CPE because one potential application of glucose biosensor is food inspection. Similar to the process in the reference [4,34,35], diluted juice samples with various standard additions of glucose were added into 0.1 M NaOH, and the corresponding amperometric responses were recorded at 0.55 V. Table 2 shows the obtained results. The biggest relative standard deviation is 3.0%, which illustrates the good reproducibility of the method [36]. The recoveries ranging from 95.2% to 100% indicate that the sensor is sufficient for analyzing glucose in food inspection application [34]. 

## 3. Materials and Methods 

Nickel nitrate hexahydrate, cerium nitrate hexahydrate, glucose, AA, UA, AP, dopamine hydrochloride, fructose, sucrose, and lactose were purchased from Sigma–Aldrich (St. Louis, MO, USA) and used without further purification. Graphite, paraffin oil, sodium hydroxide, and ammonium hydroxide were purchased from Aladdin Chemicals (Shanghai, China). 

CeO2 NRs were synthesized through a non-isothermal precipitation based on the study of Patil et al. [24]. In detail, ammonia was added (0.5 mL/min) into 100 mL 0.20 M cerium nitrate aqueous solution at 70 °C, in which a yellow precipitate was immediately formed. After 5 min, the reaction mixture was transferred into a 0 °C water bath in which the reaction continued for 24 h. Finally, the precipitate was filtered and washed with ethanol and de-ionized water and then dried at 60 °C in a vacuum. The precipitate was performed by calcination at 350 °C for 2 h under air resulting in the final CeO_2_ NRs.

CeO2/Ni(OH)_2_ nanocomposites were prepared via the wet impregnation method. Briefly, CeO_2_ NRs were dispersed in 50 mL nickel nitrate solution by magnetic stirring at 70 °C for 1 h. Thereafter, 5 mL ammonia was added (0.5 mL/min) to this solution, and a turquoise-colored precipitate was formed. Finally, this precipitate was filtered, washed three times with de-ionized water, and then dried at 60 °C in a vacuum. The amount of nickel nitrate was set as constant (10 mmol), whereas the molar ratio of cerium to nickel (Ce:Ni) was varied as 0.05:1, 0.1:1, and 0.5:1 for comparison purposes. 

XRD analysis was performed with a Y2000 diffractometer (HAOYUAN, Dandong, China) with Cu Kα radiation (λ = 1.5418 Å). TEM was conducted with a JEM2010 instrument (JEOL, Tokyo, Japan). 

The electrochemical measurements were conducted on a model CHI630D electrochemical analyzer (CHENHUA, Shanghai, China). All of the experiments were performed in 0.1 M NaOH solution at room temperature with a three-electrode electrochemical cell by using modified CPE as working electrodes, a platinum wire as a counter electrode, and an Ag/AgCl electrode as a reference electrode. 

The modified CPE working electrodes were prepared according to reference [4]. In detail, the synthesized materials, graphite powder, and paraffin oil with a mass ratio of 2:5:1 were mixed by hand in a mortar until a uniformly wetted paste was obtained. Then, this paste was filled firmly into a Teflon tube with an inner diameter of 3 mm. The electrical contact was established by pushing a copper wire down the tube into the back of paste.

## 4. Conclusions

A non-enzymatic glucose biosensor based on CeO_2_/Ni(OH)_2_ nanocomposite modified CPE was successfully developed. The enhanced sensing performance of nanocomposite was related to the synergetic effects of the two components. Specifically, Ni(OH)_2_ dominated the redox reaction of glucose by using the Ni(II)/Ni(III) redox couple, whereas the entangled CeO_2_ NRs formed conductive paths to accelerate the electron transfer to the electrode. The nanocomposite biosensor had a high sensitivity of 594 μA mM^−1^ cm^−2^, a linear range of 0.002–6.62 mM, a fast response time of less than 5 s, and an estimated detection limit of 1.13 μM (S/N = 3). The biosensor also exhibited good selectivity and stability. In addition, the results of this work clearly demonstrate that pristine CeO_2_ can be a good candidate for enhancing the performance of conventional non-enzymatic sensing materials, although it has no electrocatalytic activity and is not suitable as a non-enzymatic sensing material (e.g., CuO, NiO, Ni(OH)_2_, Cu(OH)_2_). Apart from exploring other compositions (e.g., CeO_2_/Cu(OH)_2_, CeO_2_/Co_3_O_4_) for non-enzymatic biosensor, future work should focus on optimizing the structure of these nanocomposites (for example, CeO_2_/Ni(OH)_2_ coaxial tubular nanostructure or CeO_2_/Ni(OH)_2_ hierarchical nanostructure).

## Figures and Tables

**Table 1:** Comparison of performance obtained from different glucose biosensors.

Electrode	Sensitivity (μA mM^−1^ cm^−2^)	Linearity (mM)	LOD ^c^ (μM)	Potential (V)	References
CeO_2_ NRs ^a^	0.165	2~26	100	0.80	[24]
CeO_2_/Pd ^a^	-	0.1~10	10	−0.20	[30]
CeO_2_ NPs ^a^	-	0.007~0.13	0.003	-	[31]
Ni(OH)_2_/SiMCP ^b^	250	0~8	3.5	0.50	[32]
Ni(OH)_2_-HS ^b^	223.39	0.0009~7.781	0.1	0.45	[33]
Ni(OH)_2_/TiO_2_^b^	192	0.03~14	8	0.50	[1]
Au/Ni(OH)_2_^b^	-	~2	-	0.50	[2]
Ni(OH)_2_/TiO_x_Cy ^b^	240	0.02~1.7	5.0	0.70	[9]
Ni(OH)_2_/Au ^b^	371.2	0.005~2.2	0.92	0.55	[12]
CeO_2_/Ni(OH)_2_^b^	594	0.002~6.6	1.13	0.55	This work

^a^ enzymatic biosensor; ^b^ non-enzymatic biosensor; ^c^ limit of detection (LOD).

**Table 2:** Determination of glucose in a juice on sale.

Spiked (μM)	Found (μM)	Recovery (%)	RSD (%)
0	40.3	-	-
25	64.1	95.2	2.9
50	90.3	100	2.6
75	112.7	96.5	1.9
100	135.3	95.0	3.0
